# Friction control of elastic materials on glass by means of textured surfaces

**DOI:** 10.1038/s41598-022-19338-7

**Published:** 2022-09-14

**Authors:** Naoki Fujita, Takumi Kinoshita, Masaru Iwao, Noriaki Masuda, Yoshitaka Nakanishi

**Affiliations:** 1 Research and Development Group, Nippon Electric Glass Co., Ltd., 2-7-1 Seiran, Otsu, Shiga 520-8639 Japan; 2 Graduate School of Science and Technology, Kumamoto University, 2-39-1 Kurokami, Chuo-ku, Kumamoto 860-8555 Japan; 3 Faculty of Advanced Science and Technology, Kumamoto University, 2-39-1 Kurokami, Chuo-ku, Kumamoto 860-8555 Japan

**Keywords:** Engineering, Mechanical engineering, Applied physics

## Abstract

To investigate the friction behaviors of elastomer and polyacetal writing tips sliding on various textured glass surfaces, the influences of the pitch size and height of sub-millimeter to millimeter sized texture on friction were examined via reciprocating friction tests. The friction coefficients of each writing tip could be systematically varied by changing the pitch and height of the texture. These changes in friction were based on the relationship between the convex-concave shapes and the contact parts of the writing tip, and hence, influence the adhesive, abrasive, and deformation frictions. By inducing a surface texture with a pitch smaller than the contact area of the writing tip, the friction coefficient could be reduced effectively. By inducing a surface texture with a larger height, the friction coefficient of the elastomer could be increased due to deformation friction. These behaviors indicate the possibility of controlling the friction by changing the parameters such as the pitch and height of the textured glass surfaces.

## Introduction

Controlling tribological properties using textured surfaces has been attractive in a variety of fields to improve sliding surface conditions^1–4^. For instance, in the field of automobiles, textured surfaces have been introduced to control friction and improve energy efficiency, which in turn, reduces CO_2_ emissions^5,6^. Improving the tribological properties of machine tools has also been investigated by introducing texture on the surfaces of machine tool guideways, journal bearings, and other components^7–9^.

Numerous studies have examined the effects of textured surfaces on the tribological characteristics under both lubricated and unlubricated conditions^10–14^. Under lubricated conditions, textured surfaces generally enhance hydrodynamic lubrication^15,16^. In contrast, under unlubricated conditions, textured surfaces contribute to apparent contact areas, which also significantly affects the adhesive friction and abrasive friction at the asperity level of counterface^17–20^. Moreover, deformation friction of an elastic material due to a textured surface can also affect friction behaviors^21–24^. The elastic deformation and migration of the elastic material moving along the convex and concave structure in the textured hard counterface may cause fatigue wear of the elastic material^25^.

From the standpoint of reducing paper use and improving work efficiency, handwriting input on tablet computers has become more widespread in recent years^26^. In the handwriting input, the frictional behaviors of pen tips, which influence the writing experience, are critical^27^. Users typically have a poor experience writing with a pen on a slippery flat glass surface. Common methods to improve the user experience include increasing the friction by adjusting the elastic modulus or changing the material of the pen tip and attaching a textured film sheet onto the tablet computers. Introducing textures directly on the glass surfaces of tablet computers has the advantages of preserving the scratch resistance, transparency, and the feel of the glass and therefore, has attracted considerable attention. However, there are only a few studies that aim to improve the handwriting input experience by introducing textures on the glass. In a previous study, we reported on the possibility of controlling the frictional characteristics using two types of surface roughness (sub-millimeter to millimeter sized texture and nanometer sized fine roughness)^28^. We found that each roughness affects the adhesive friction, abrasive friction, and deformation friction, and therefore, the coefficient of friction between the writing tips and the textured glass surfaces can be controlled. However, a discussion on the contribution of pitches and heights of larger sized convex and concave shapes to the frictional behaviors was lacking.

In this study, we measured the friction behaviors of two types of commercially available pen tips on textured glass by changing the condition of the roughness in detail. The friction behaviors were observed via reciprocating friction tests in relation to textured glass surfaces with two types of roughness: The first type is due to nanometer sized asperities; this kind of roughness can influence adhesive and abrasive frictions. The other is due to surface texture of sub-millimeter to millimeter sized pitches and can influence deformation. We focused particularly on the pitch size and height of sub-millimeter to millimeter sized texture. The frictional mechanisms based on adhesive, abrasive, and deformation frictions were analyzed based on the relationship between the sub-millimeter to millimeter sized texture and changes in friction.

## Methods

### Writing tip

The writing-tip specimens used in this study were an elastomer tip (ACK-20004, Pen Nibs, Wacom Co., Ltd.) and a polyacetal tip (ACK-20001, Pen Nibs, Wacom Co., Ltd.) (Supplementary Fig. S1). The elastomer tip consists of thermoplastic polyester elastomer, which has a structure with slits. The surface roughness (Sa) and radius of curvature at the tip of the elastomer writing-tip specimen were approximately 10.5 μm and 687 μm, respectively, and those of the polyacetal tip were approximately 0.7 μm and 665 μm, respectively.

### Preparation of textured glass surface for writing

The processing of the textured glass surface was realized via micro-slurry-jet processing (Supplementary Fig. S2)^29–31^. Flat glass plates with dimensions of 70 mm (length) × 70 mm (breadth) × 0.55 mm (thickness) (flat glass plate; T2X-1, Nippon Electric Glass Co. Ltd., Japan) were used. A slurry consisting of pure water with 3 wt% alumina particles (WA # 8000, average diameter = 1.2 μm) was prepared. The slurry was sprayed vertically on the glass surfaces through a 1 mm-wide square-shaped injection nozzle using compressed air at 0.23 MPa. The injection nozzle could be moved parallel to the processed surface using a numerical control system. Textured surfaces were prepared by adjusting the speed and pitch of the nozzle. A non-processed glass plate (flat glass plate) was also used for frictional testing.

The glass surfaces were analyzed using a three-dimensional optical surface profiler (NewView 7300, Zygo Co., USA). The geometrical parameters of the textured glass surface, which exhibits both convex and concave sections, were defined from the 3D bird’s-eye view and line profile (Supplementary Fig. S3). The figure also shows the pitch between the concave parts and the height between the convex and concave parts. We measured the surface roughness (Sa) across a 75 μm × 55 μm area of the convex section (Supplementary Fig. S3). The measured Sa values in the micro region varied with the measurement points; hence, the average of measurement results at any 10 points was calculated.

### Measurement of friction coefficient via reciprocating motion tests

Friction was measured by conducting reciprocating motion tests (Supplementary Fig. S4). During the test, the writing tip was fixed at an orientation angle of 60° and pressed onto the glass plate under a load of 0.98 N and 1.96 N. Reciprocating motions with a stroke of 50 mm and a speed of 5 mm/s were applied for 100 cycles at room temperature (approximately 25 °C). The coefficient of friction was calculated from the frictional force measured by a load cell. The writing tip was replaced after each test. To stabilize the surface condition before testing, the glass writing specimen was ultrasonically cleansed three times with ultrapure water and once with ethyl alcohol and was then set aside for 5 days.

### Contact area observations between glass surface and writing tip during sliding motion

The contact area between the writing tips and a glass surface during sliding motion was observed (Supplementary Fig. S5). During the observations, the writing tips were fixed at an orientation angle of 60° and pressed onto the glass plate under a load of 1.96 N. The contact area between the writing tips and the glass surface was observed from the reverse side of the glass using a laser microscope (LEXT OLS5000-SAT, Olympus Corporation, Japan). A reciprocating motion in the right and left direction, with a stroke of 12 mm and a speed of 1 mm/s, was induced by operating an x-stage micrometer with a stepping motor; the glass specimens on the x-stage were moved against fixed writing tips. To reduce the influence of the vibration of the device on the observation, test was conducted at a speed of 1 mm/s, which is slower than the reciprocating motion tests.

## Results and discussion

### Surface properties of textured glass

**Figure 1:**
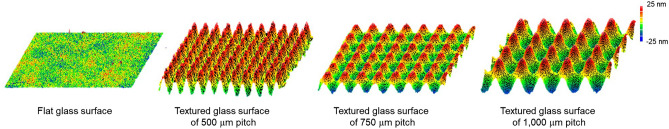
Images of glass surfaces considered as writing-surface specimens (flat glass, pitch of 500 μm, 750 μm, and 1000 μm).

Table 1 shows the surface parameters of the glass surfaces used as the writing-surface specimens. Figure 1 shows example observation images of flat glass and each pitch (height: 21.6–23 nm). The formation of continuous and uniform concave-convex shapes can be seen on the textured glass surfaces. Moreover, all processed glass remained transparent^32^. In all pitches, Sa tends to increase with an increase in height between the convex and concave parts due to the increased collision rate of the alumina particles at each point. These increases in the Sa and height were based on feeding speed of the injection nozzle of the micro-slurry-jet processing; that is, the lower feeding speed, the rougher the Sa and the greater the height of the convex and concave shapes.

**Table 1:** Surface parameters of glass surfaces considered as writing-surface specimens.

Flat glass		Height, nm Roughness (Sa), nm	− 0.09						
Pitch	500 μm	Height, nm Roughness (Sa), nm	1.7 2.33	3.1 3.13	5.5 4.10	8.8 5.22	21.6 6.23	45.1 6.81	
	750 μm	Height, nm Roughness (Sa), nm	0.9 1.99	3.5 2.73	4.5 3.47	6.9 4.02	14.2 5.09	21.6 5.77	35.3 6.27
	1000 μm	Height, nm Roughness (Sa), nm	4.3 1.66	9.1 1.97	13.6 2.69	23.8 3.15	49.0 4.63

### Influence of height difference on friction behaviors for each pitch

**Figure 2:**
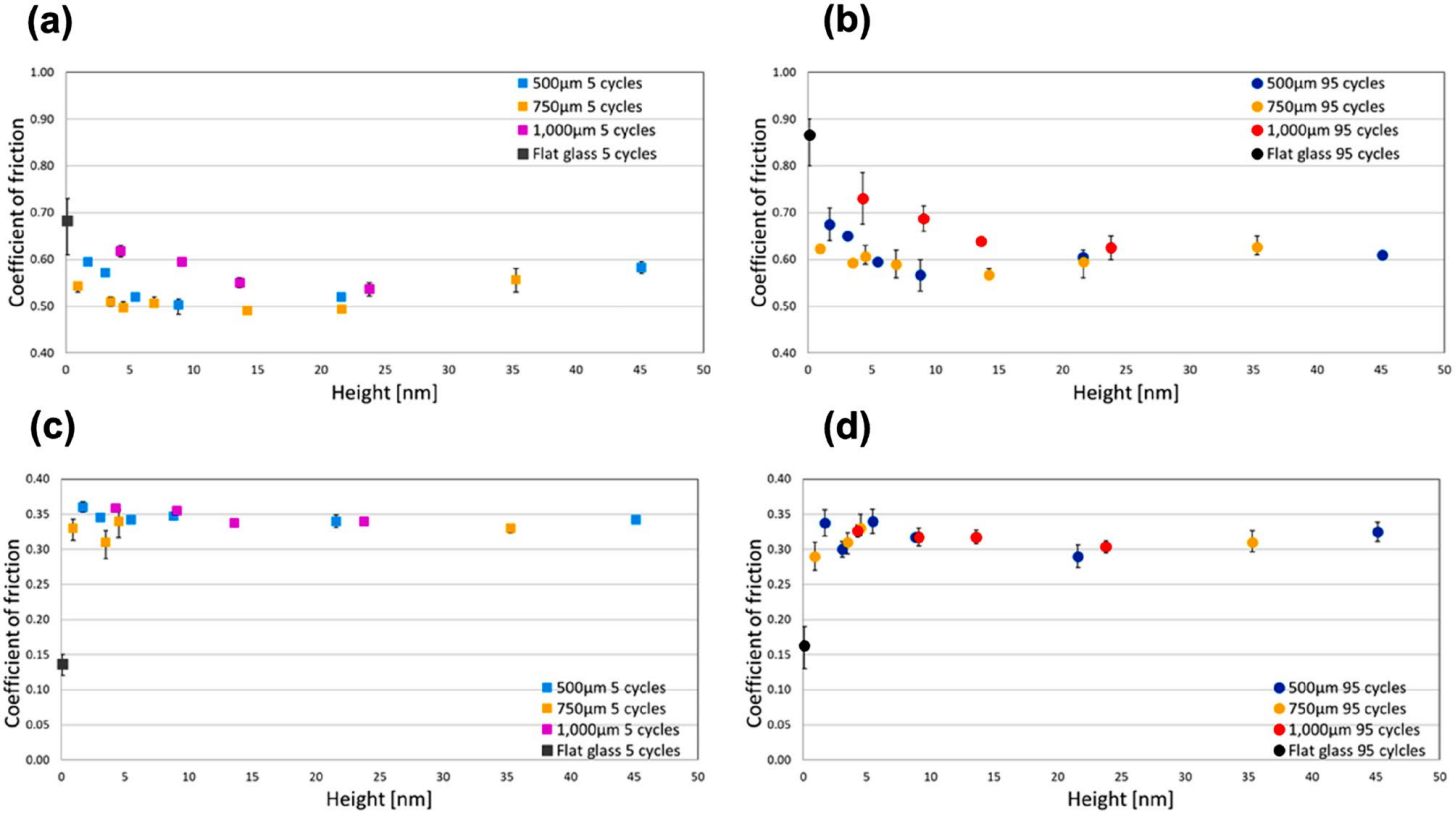
Relation between the friction coefficient and the convex-concave height in the glass surfaces for the elastomer and polyacetal tips. (**a**) Elastomer after 5 cycles; (**b**) Elastomer after 95 cycles; (**c**) Polyacetal after 5 cycles, (**d**) Polyacetal after 95 cycles.

Figure 2 shows the experimental results of reciprocating friction tests. The friction measurements for the two types of tips and the various surfaces were conducted two or three times, and the mean friction coefficient after the 5th (Fig. 2a and c) and 95th (Fig. 2b and d) cycles was recorded^28^. For the elastomer tip, the friction increased with the number of reciprocation cycles for all samples (Fig. 2a and b). These results indicate elastomer damage; the presence of transferred elastomer on the glass surface can increase the friction with the number of reciprocation cycles^33–35^. For the polyacetal tip, friction tended to decrease marginally with the number of reciprocation cycles (Fig. 2c and d). The same tendency was seen in all glass surfaces; therefore, the formation of transferred polyacetal layer on the glass surfaces is considered to contribute to the reduction in the friction coefficient^36–39^. Additionally, the surface properties and the real contact at the asperity level of glass surfaces are crucial to the frictional behaviors of the writing tips and the glass surfaces. Nanometer sized asperities on the glass surfaces influence friction, leading to different friction behaviors of the elastomer and polyacetal. Furthermore, because the surface roughness of each writing tip was much higher than that of the nanometer sized asperity on the glass surfaces, the dimensions of the nanometer sized asperity had little effect on friction^28^. The distinctions between the frictional behaviors among the pitches are evident in Fig. 2. In the elastomer (Fig. 2a and b), the friction coefficient of the flat glass surface was the highest because of the high adhesion force of the elastomer^40,41^. Inducing surface textures on the flat glass surface resulted in a decrease in the friction coefficient of all textured surfaces owing to the reduced contact area between the elastomer and glass surfaces. That is, it can be considered that the amount of adhesive friction decreased due to the decrease in the contact area by nanometer sized asperity and sub-millimeter to millimeter sized texture^28,42^. The coefficients of friction of the 750 μm pitches dramatically decreased with an increase in the height of the convex and concave shapes. The reduction in the coefficients of friction of the 1000 μm pitches was the slowest among the three pitches. The coefficients of friction of the 500 μm pitches showed an intermediate behavior. These results can be interpreted based on the relation between the diameter of the contact area of the elastomer tip (approximately 850 μm) and glass surfaces^28^. The 750 μm pitches are smaller than and closer to the diameter of the contact area (850 μm), which might have a significant role in reducing the apparent contact area between the elastomer and the glass surfaces. In contrast, in case of the 1000 μm pitches, the elastomer can easily reach the concave part of the texture because the pitch size is larger than the diameter of the contact area; hence, the reduction in the apparent contact area is negligible. In case of the 500 μm pitches, although the effect of apparent contact area was smaller than that in the 750 μm pitches, a similar coefficient of friction can be obtained as the height of the convex and concave increases (~ approximately 20 nm). When the height of the convex and concave is greater than or equal to 20 nm, the coefficient of friction tends to increase. This increase in the coefficient of friction at a greater height may be due to deformation friction resulting from the elastic deformation when the elastomer enters the concave part^42–45^. The balance of friction coefficient around 20 nm height between 500 and 750 μm pitches is assumed to depend on the degree of deformation friction, which implies that it is easier for the elastomer to enter the concave parts of the 750 μm pitches. At 1000 μm pitches, the elastomer is more likely to enter the concave parts than other pitches, hence reduction in the friction coefficient was gradual. In case of polyacetal (Fig. 2c and d), the friction coefficient of flat glass was the lowest among the glass surfaces due to low adhesive friction of the polyacetal. As the nanometer sized asperity caused abrasive friction on the surfaces, the friction coefficient of the textured glass surfaces increased compared with that of the flat glass^46–48^. In all pitches, a larger height tends to correspond to a lower friction coefficient. There was no significant difference in the friction behaviors between the pitches. In a previous study, the diameter of the contact area of the polyacetal tip and glass surfaces was determined to be approximately 180 μm^28^. Therefore, the polyacetal tip could reach the concave parts of the texture as all the pitch sizes were larger than the contact area. However, when the pitch size is large (1000 μm), it is easier for the polyacetal tip to enter into the concave parts. Therefore, the friction coefficients of the 1000 μm pitches were slightly higher than those of other pitches. When the height of the convex and concave is larger, it is assumed that, during sliding motions, the polyacetal tip cannot reach the bottom of the concave parts of all pitches. Consequently, the contact area between the polyacetal and the glass surfaces is reduced and this contributes to a decrease in the frictional coefficient of the textured glass surfaces.

**Figure 3:**
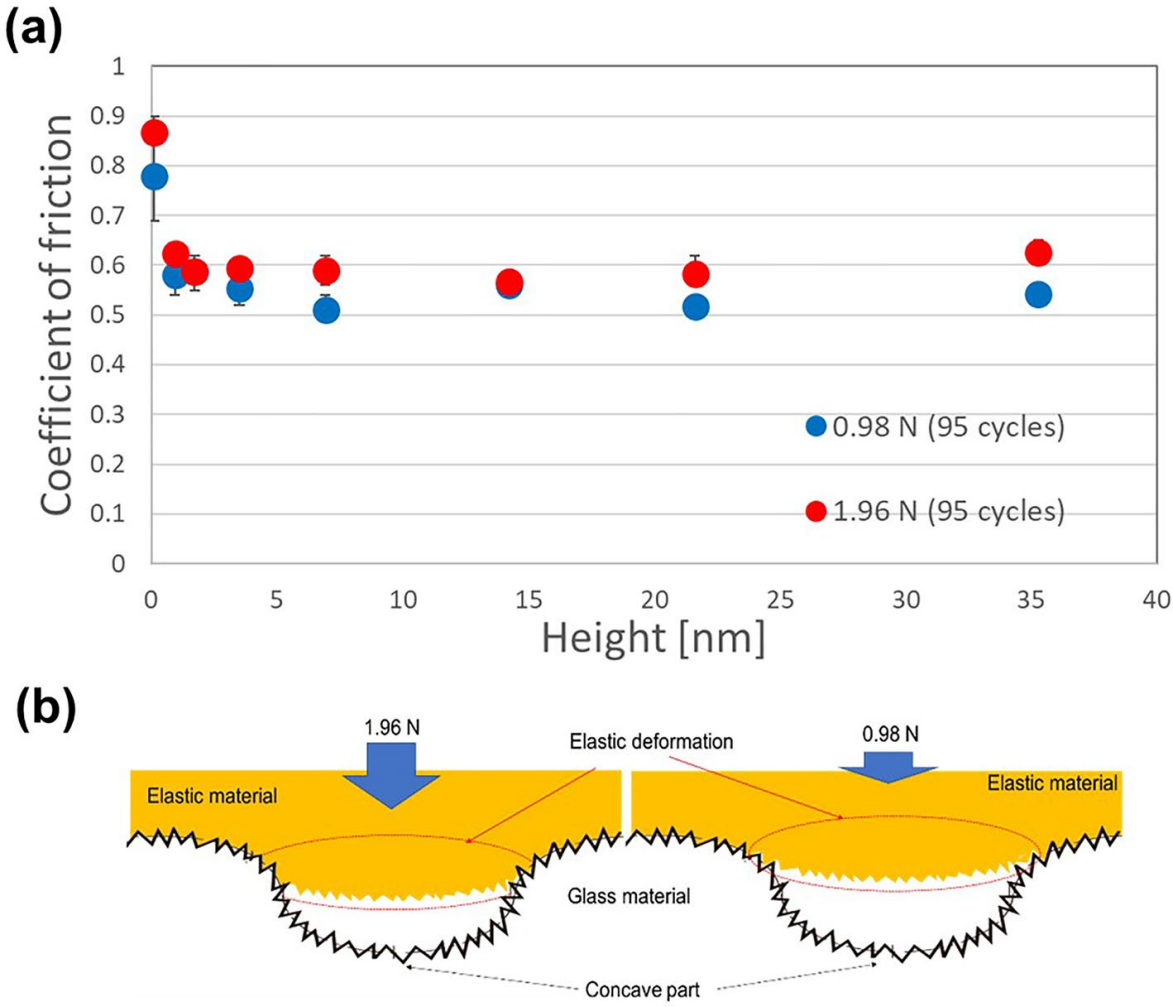
Difference in friction behavior in 750 μm pitches under the loads of 1.96 N and 0.98 N. (**a**) Relation between the friction coefficient and the convex-concave height in the glass surfaces for the elastomer under 1.96 N and 0.98 N of load. (**b**) Images of difference in the amount of deformation under 1.96 N and 0.98 N of load.

To investigate the influence of deformation friction in the elastomer when the height of the convex and concave is large, the applied load in the reciprocating motion test in 750 μm pitches was changed from 1.96 N into 0.98 N. A smaller applied load is expected to reduce the amount of deformation of the elastomer into the concave parts. Figure 3a shows the experimental result (the friction coefficient of 95th cycles) at applied loads of 1.96 N and 0.98 N load for the 750 μm pitches. Although the behavior of friction under 1.96 N and 0.98 N up to approximately 22 nm of height was similar, a difference was observed around 35 nm of height. When the applied load was 0.98 N, the friction coefficient did not increase in even at a large height of the convex and concave parts due to reduced deformation friction. Accordingly, the increase in height of the convex and concave parts is suggested to increase the deformation friction caused by the writing tip entering the concave parts (Fig. 3b). The frictional behaviors were determined from the friction measurement results and the geometrical factors due to the contact between the glass surfaces and writing tips. To understand these behaviors, the frictions must be interpreted on the basis of the contact mechanism between the glass surfaces and the writing tips. Although fundamental contact mechanics theories based on real contact area have been developed in detail^49–53^, the contact state between the glass, containing two types of rough surfaces with considerably different roughness values, and the writing tip with rough surfaces must be accounted for in this study. Therefore, in the future, we plan to determine the mechanism of the phenomenon on a scientific basis, via contact mechanics.

### Observation results of contact area between the writing tips and glass surface during sliding motion

**Figure 4:**
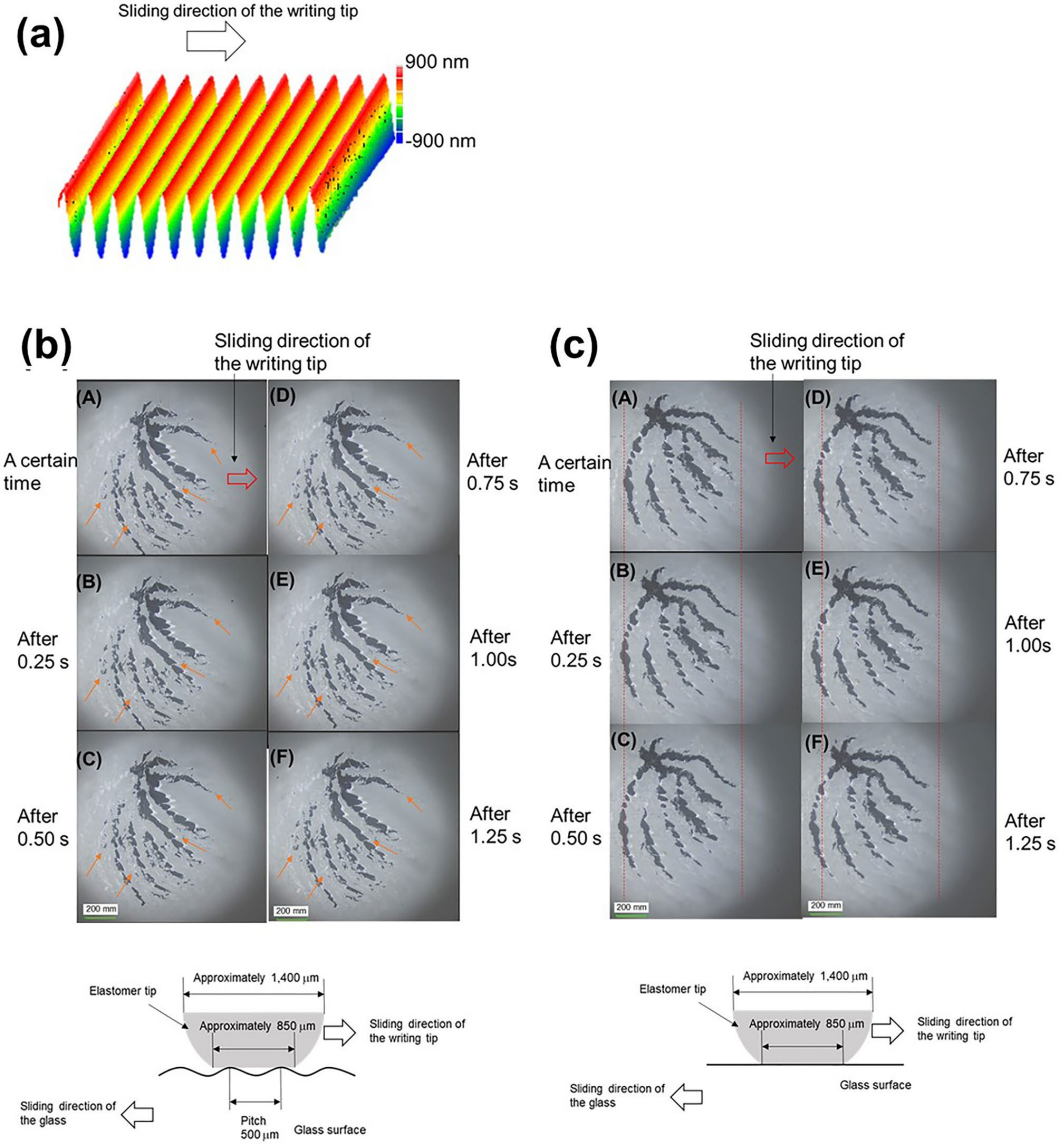
(**a)** Stripe pattern with 500 μm spacing and 1463 nm of the height and sliding direction of the writing tips. (**b**),(**c**) Observations of the contact area between the elastomer and glass surfaces during sliding motion ((**b**) Stripe pattern with 0.5 mm spacing and 1463 nm, (**c**) flat glass), and illustration of the relationship between the elastomer and each glass surface.

**Figure 5:**
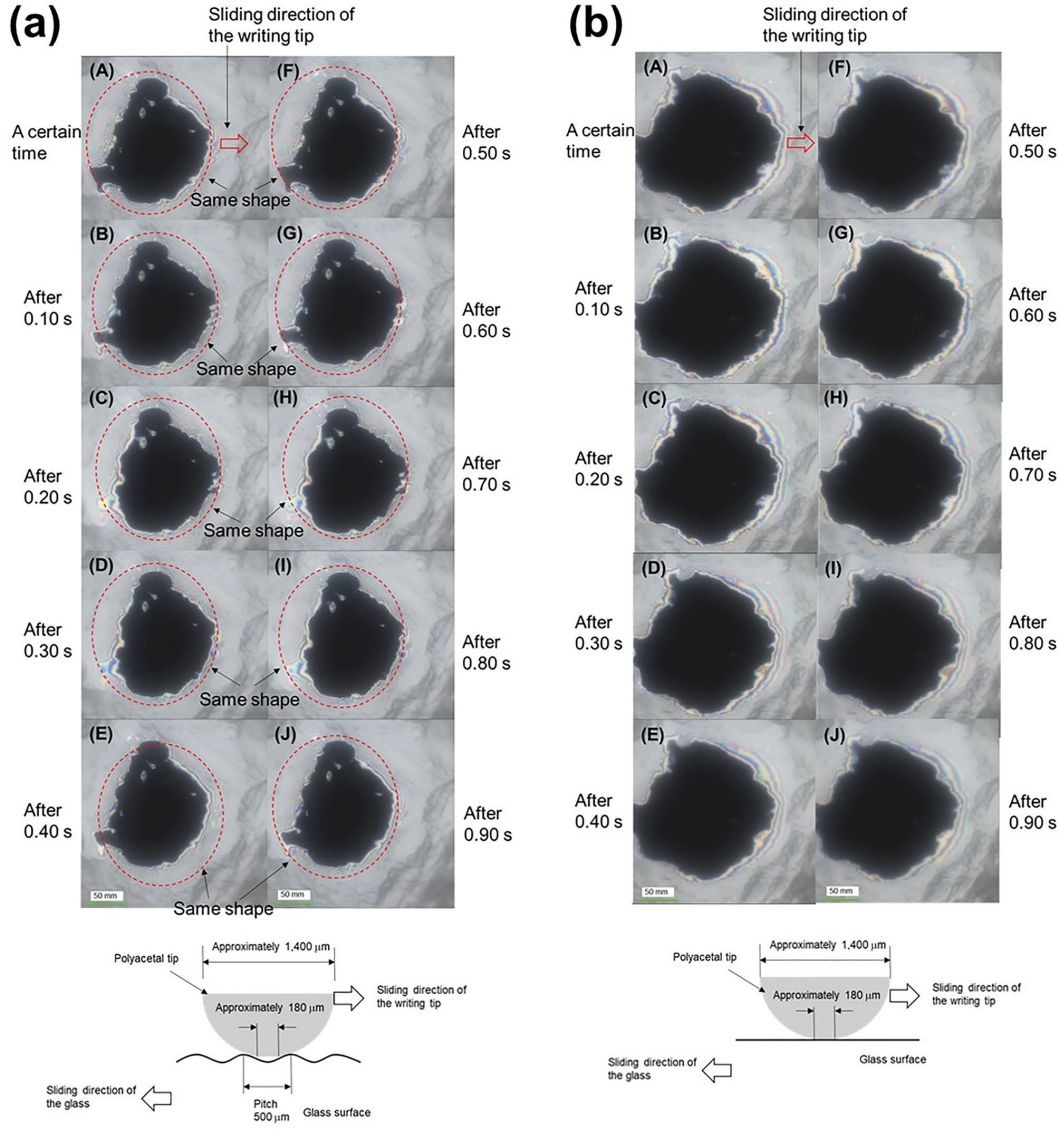
Observations of the contact area between the polyacetal and glass surfaces during sliding motion ((**a**) Stripe pattern with 0.5 mm spacing and 1463 nm, (**b**) flat glass), and illustration of the relationship between the polyacetal and each glass surface.

**Figure 6:**
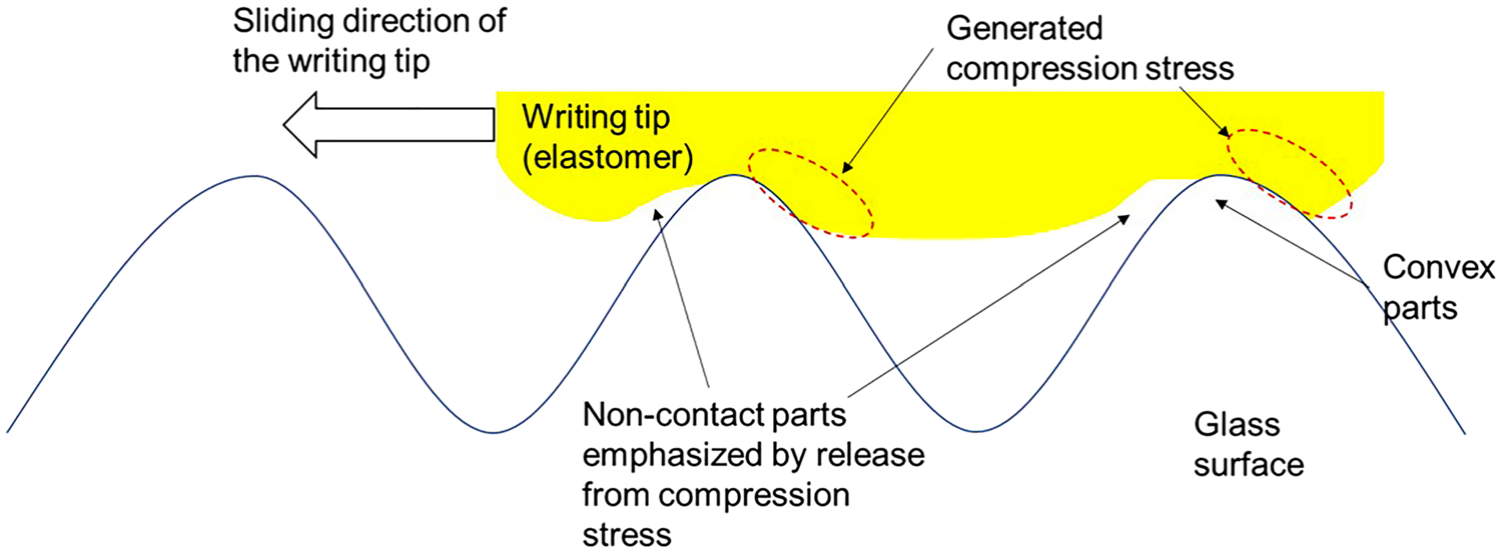
Illustration of the non-contact parts emphasized after being released from compression stress near convex parts for the elastomer.

**Figure 7:**
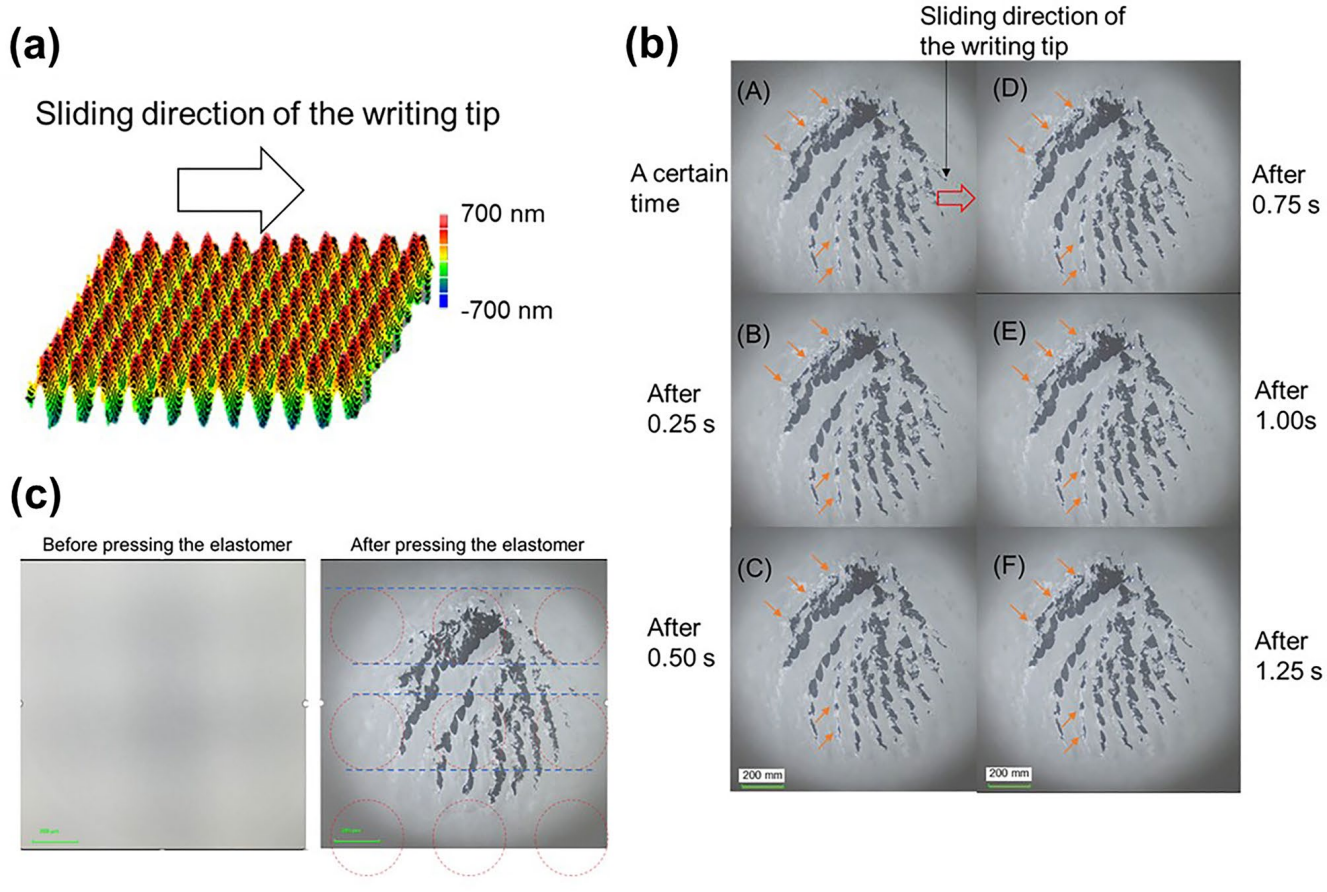
(**a**) Lattice-shaped pattern with 500 μm spacing and 560 nm of the height and sliding direction of the writing tips. (**b**) Observations of contact area between the elastomer and lattice-shaped patterned glass surfaces during sliding motion (pitch: 500 μm, height: 560 nm). (**c**) Observation of the contact part before and after pressing the elastomer onto lattice-shaped patterned glass surface (pitch: 500 μm, height: 560 nm). The lattice-shaped pattern can be observed before pressing the elastomer onto the glass surface.

To examine the relation between the changes in the friction coefficient and the sub-millimeter to millimeter sized texture, observation of the behaviors at the contact region during the sliding motions is crucial. However, an observation of the minute changes at the contact region is difficult because the height of the convex and concave is quite low (~ 50 nm). Therefore, the observations during sliding motions were conducted using a sample with an enhanced height (~ 1463 nm) and stripe pattern with 500 μm spacing; the glass surfaces were moved in a direction perpendicular to the stripe pattern against the fixed writing tips (Fig. 4a). Figures 4b,c and 5a,b and Supplementary Videos 1–2 show the observation results of the stripe textured surface (Figs. 4b, 5a) and the flat glass surface (Figs. 4c, 5b) in elastomer and polyacetal. From a video recording of the observation, an image was extracted every 0.25 s for elastomer (Fig. 4b and c: (A)–(F)) and every 0.10 s for polyacetal (Fig. 5a and b: (A)–(J)). For the textured surface in the elastomer (Fig. 4b), the change in the shape at the contact part was significantly small, and regular change at the contact part (fluctuations between contact and non-contact parts in the contact part) could be confirmed. The fluctuations are changes wherein the contact part becomes smaller, larger, appears, or disappears (the parts indicated by the orange arrows), and can be observed in a 0.5 s cycle (Fig. 4b). Furthermore, the fluctuations appear to move against the sliding direction of the elastomer tip. The 0.5 s corresponds to the cycle moving 0.5 mm at a speed of 1 mm/s (sliding speed of the writing tip). Thus, these fluctuations can indicate the non-contact parts of the elastomer above the concave parts of glass surface. These fluctuations can be clearly seen in the video. On the contrary, for the flat glass surface in the elastomer (Fig. 4c), regular change in the contact part was not observed. Gradual change in the shape at the contact part and a shift to the left side were confirmed; this can be attributed to the high adhesion of the elastomer (The shift from the red dotted line is confirmed). Figure 6 shows an image of the contact part between the elastomer tip and the convex and concave. When the writing tip is in contact with the convex part, a compression stress is generated near the slope of convex part of the writing tip. Once the writing tip moves away from the convex part, the generated compression stress is released, and the non-contact parts are emphasized in the observation images. In this study, although only non-contact parts next to the released area from compression stress were observed clearly, it is considered that the concave parts were not in contact with the writing tip. For the polyacetal, the fluctuation of 0.50 s cycle could be observed clearly in the textured surface compared with flat glass surface (Fig. 5a and b). In the textured surface, it can be clearly observed that the shape of the contact part corresponds to that after 0.50 s. Since polyacetal is a harder material than elastomer, it is hard to absorb the fluctuations in vibration; hence, observation of the release of compression stress is easier. The observation results of the lattice-shaped pattern (pitch: 500 μm, Height: 560 nm (Fig. 7a) for the elastomer is shown in Fig. 7b and Supplementary Video 3. Figure 7c shows a contact condition between the elastomer tip and convex parts of the textured surface. Similar fluctuations of 0.50 s cycle were observed in a few portions of the contact part. These fluctuation parts correspond to the convex parts (Fig. 7c). The fluctuations were mainly observed within the range of the blue dotted line that coincides with the convex part in Fig. 7c (the parts indicated by the orange arrows in Fig. 7b). Therefore, the non-contact parts of the elastomer tip are suggested to exist after passing through the convex parts. Although these observations were conducted on textured surfaces with enhanced height to emphasize the phenomenon, we believe that similar behavior near the convex parts occurs at a low height of the convex and concave shapes.

Inducing two types of surface roughness on the glass surface has an interesting effect on the frictional characteristics. In particular, this study showed that the friction coefficient can be modified by controlling the size of sub-millimeter to millimeter sized texture after understanding the role of each roughness. The control of fiction characteristics demonstrated in this study can open new avenues in various fields apart from improving the user experience of pen-input devices.

## Conclusions

We investigated the friction behavior of elastic materials on textured glass surfaces via reciprocating friction tests. These tests were conducted using two types of writing tips (elastomer and polyacetal) and textured glass surfaces prepared via micro-slurry-jet technology. The textured glass surfaces have sub-millimeter to millimeter sized texture and fine roughness at the nanometer scale, which result in friction behavior difference arising from adhesive, abrasive, and deformation frictions. In this study, we specifically focused on height and pitch size of sub-millimeter to millimeter sized texture, which can affect the friction characteristics. We observed the differences in the influence of height and pitch size of sub-millimeter to millimeter sized concave-convex surfaces on the friction behaviors. The significant conclusions are:

1. For the elastomer, the relation between the apparent contact area of the writing tip and the concave-convex surfaces for reduction of the friction coefficient: when the pitch size is smaller than the diameter of the contact part, the friction coefficient tends to decrease with respect to the height of the concave-convex. When the pitch size is larger than that, the reduction in the friction coefficient decreases, and a larger height of the concave-convex is required to reduce the friction coefficient.
2. Increase in the friction coefficient at a larger height of the concave- convex (pitch: 750 μm, height: 35 nm) is due to deformation friction caused by the writing tip entering the concave parts.
3. For the polyacetal, as the contact area between the writing tip and the concave-convex surfaces is smaller than all pitches, the contribution from the pitch size is small. When the pitch size is much larger than the contact area (1000 μm), the writing tip is more susceptible to abrasive friction on nanometer sized asperities and has a higher friction coefficient than other pitches (500 μm and 750 μm).
4. A clue concerning the existence of the non-contact part immediately after passing through convex parts could be obtained from the observations of the contact part during sliding motions.

We expect that our findings can accelerate development to improve the user experience of pen-input devices.

## Supplementary Information

Supplementary Information 1.

Supplementary Information 2.

Supplementary Video 1.

Supplementary Video 2.

Supplementary Video 3.

## Data Availability

The datasets generated during and analyzed during the current study available from the corresponding author on reasonable request.

## References

[1] Wandersman E, Candelier R, Debrégeas G, Prevost A (2011). Texture-induced modulations of friction force: the fingerprint effect. Phys. Rev. Lett..

[2] Li J, Xiong D, Dai J, Huang Z, Tyagi R (2010). Effect of surface laser texture on friction properties of nickel-based composite. Tribol. Int..

[3] Yu C, Wang QJ (2012). Friction anisotropy with respect to topographic orientation. Sci. Rep..

[4] Khojasteh B, Janko M, Visell Y (2018). Complexity, rate, and scale in sliding friction dynamics between a finger and textured surface. Sci. Rep..

[5] Holmberg K, Erdemir A (2019). The impact of tribology on energy use and CO2 emission globally and in combustion engine and electric cars. Tribol. Int..

[6] Holmberg K, Andersson P, Erdemir A (2012). Global energy consumption due to friction in passenger cars. Tribol. Int..

[7] Nakano M (2007). Applying micro-texture to cast iron surfaces to reduce the friction coefficient under lubricated conditions. Tribol. Lett..

[8] Wang L, Guo S, Wei Y, Yuan G, Geng H (2019). Optimization research on the lubrication characteristics for friction pairs surface of journal bearings with micro texture. Meccanica.

[9] Reinert L (2017). Long-lasting solid lubrication by CNT-coated patterned surfaces. Sci. Rep..

[10] Ronen A, Etsion I, Kligerman Y (2001). Friction-reducing surface-texturing in reciprocating automotive components. Tribol. Trans..

[11] Krupka I, Hartl M, Zimmerman M, Houska P, Jang S (2011). Effect of surface texturing on elastohydrodynamically lubricated contact under transient speed conditions. Tribol. Int..

[12] Mourier L, Mazuyer D, Ninove F-P, Lubrecht AA (2010). Lubrication mechanisms with laser-surface-textured surfaces in elastohydrodynamic regime. Proc. Inst. Mech. Eng. Part J J. Eng. Tribol..

[13] Xu X, Li Y, Wu QMJ (2020). A completed local shrinkage pattern for texture classification. Appl. Soft Comput..

[14] Vlădescu S-C, Olver AV, Pegg IG, Reddyhoff T (2016). Combined friction and wear reduction in a reciprocating contact through laser surface texturing. Wear.

[15] Etsion I (2013). Modeling of surface texturing in hydrodynamic lubrication. Friction.

[16] Peng Y (2021). Elastohydrodynamic friction of robotic and human fingers on soft micropatterned substrates. Nat. Mater..

[17] Flegler F, Neuhäuser S, Groche P (2020). Influence of sheet metal texture on the adhesive wear and friction behaviour of EN AW-5083 aluminum under dry and starved lubrication. Tribol. Int..

[18] Zou M, Cai L, Wang H (2006). Adhesion and friction studies of a nano-textured surface produced by spin coating of colloidal silica nanoparticle solution. Tribol. Lett..

[19] Jourani A, Dursapt M, Hamdi H, Rech J, Zahouani H (2005). Effect of the belt grinding on the surface texture: Modeling of the contact and abrasive wear. Wear.

[20] Shibata K, Nakashima Y, Nakanishi Y (2019). Anti-fingerprint glass surface created by mechanical removal process. J. Biomed. Sci. Eng..

[21] Hu J, Song H, Sandfeld S, Liu X, Wei Y (2021). Multiscale study of the dynamic friction coefficient due to asperity plowing. Friction.

[22] Mishra T, de Rooij M, Shisode M, Hazrati J, Schipper DJ (2020). A material point method based ploughing model to study the effect of asperity geometry on the ploughing behaviour of an elliptical asperity. Tribol. Int..

[23] Bhushan B, Nosonovsky M (2004). Comprehensive model for scale effects in friction due to adhesion and two- and three-body deformation (plowing). Acta Mater..

[24] Liu Z, Sun J, Shen W (2002). Study of plowing and friction at the surfaces of plastic deformed metals. Tribol. Int..

[25] Wu C (2021). Surface texture transfer in skin-pass rolling with the effect of roll surface wear. Wear.

[26] Lyu B, Lai C, Lin C-H, Gong Y (2021). Comparison studies of typing and handwriting in Chinese language learning: a synthetic review. Int. J. Educ. Res..

[27] Gerth S (2016). Adapting to the surface: a comparison of handwriting measures when writing on a tablet computer and on paper. Hum. Mov. Sci..

[28] Fujita N, Yamaguchi H, Kinoshita T, Iwao M, Nakanishi Y (2022). Friction behaviors of elastic materials sliding on textured glass surfaces. Tribol. Int..

[29] Nakanishi Y, Nakashima Y, van der Heide E (2021). Microstructuring glass surfaces using a combined masking and microslurry-jet machining process. Precis. Eng..

[30] Baba T (2019). Micro-slurry jet for surface processing of dental ceramics. Biosurf. Biotribol..

[31] Yin L, Baba T, Nakanishi Y (2018). Fracture-free surfaces of CAD/CAM lithium metasilicate glass-ceramic using micro-slurry jet erosion. J. Mech. Behav. Biomed. Mater..

[32] Roumili F, Benbahouche S, Sangleboeuf J-C (2015). Mechanical strength of soda-lime glass sandblasted by gravitation. Friction.

[33] Kasem H (2019). Rubber plunger surface texturing for friction reduction in medical syringes. Friction.

[34] Gachot C, Rosenkranz A, Buchheit R, Souza N, Mücklich F (2016). Tailored frictional properties by Penrose inspired surfaces produced by direct laser interference patterning. Appl. Surf. Sci..

[35] Muhr AH, Roberts AD (1992). Rubber abrasion and wear. Wear.

[36] Samyn P, Schoukens G, Quintelier J, De Baets P (2006). Friction, wear and material transfer of sintered polyimides sliding against various steel and diamond-like carbon coated surfaces. Tribol. Int..

[37] Mergler YJ, Schaake RP, Huisint Veld AJ (2004). Material transfer of POM in sliding contact. Wear.

[38] Pogačnik A, Kalin M (2012). Parameters influencing the running-in and long-term tribological behaviour of polyamide (PA) against polyacetal (POM) and steel. Wear.

[39] Tanaka K, Miyata T (1977). Studies on the friction and transfer of semicrystalline polymers. Wear.

[40] Chernyak YuB, Leonov AI (1986). On the theory of the adhesive friction of elastomers. Wear.

[41] Kendall K (1975). Rolling friction and adhesion between smooth solids. Wear.

[42] Tiwari A, Tolpekina T, van Benthem H, Gunnewiek MK, Persson BNJ (2021). Rubber adhesion and friction: Role of surface energy and contamination films. Front. Mech. Eng..

[43] Mahdi D, Riches A, Gester M, Yeomans J, Smith P (2015). Rolling and sliding: Separation of adhesion and deformation friction and their relative contribution to total friction. Tribol. Int..

[44] Moore DF, Geyer W (1974). A review of hysteresis theories for elastomers. Wear.

[45] Popov VL, Dimaki A, Psakhie S, Popov M (2015). On the role of scales in contact mechanics and friction between elastomers and randomly rough self-affine surfaces. Sci. Rep..

[46] Goddard J, Wilman H (1962). A theory of friction and wear during the abrasion of metals. Wear.

[47] Kato K (1997). Abrasive wear of metals. Tribol. Int..

[48] Stupkiewicz S, Mróz Z (1999). A model of third body abrasive friction and wear in hot metal forming. Wear.

[49] Fischer-Cripps AC (1999). The Hertzian contact surface. J. Mater. Sci..

[50] Persson BNJ (1998). On the theory of rubber friction. Surf. Sci..

[51] Persson BNJ (2006). Contact mechanics for randomly rough surfaces. Surf. Sci. Rep..

[52] Persson BNJ (2001). Theory of rubber friction and contact mechanics. J. Chem. Phys..

[53] Persson BNJ, Tosatti E (2001). The effect of surface roughness on the adhesion of elastic solids. J. Chem. Phys..

