# Supplementary material for: Friction control of elastic materials on glass by means of textured surfaces
Source: Sci Rep. 2022 Sep 14;12:15423. doi: 10.1038/s41598-022-19338-7 (PMC9474819; doi:10.1038/s41598-022-19338-7)
Supplement: Supplementary file 1 — Supplementary Information 1. [file 41598_2022_19338_MOESM1_ESM.pdf]

Friction control of elastic materials on glass by means of textured surfaces

Naoki Fujita[1,2,*], Takumi Kinoshita[1], Masaru Iwao[1], Noriaki Masuda[1], and Yoshitaka Nakanishi[3]

[1] Research and Development Group., Nippon Electric Glass Co., Ltd., 2-7-1 Seiran, Otsu, Shiga 520-8639, Japan

[2] Graduate School of Science and Technology, Kumamoto University, 2-39-1 Kurokami, Chuo-ku, Kumamoto 860-8555, Japan

[3] Faculty of Advanced Science and Technology, Kumamoto University, 2-39-1 Kurokami, Chuo-ku, Kumamoto 860-8555, Japan

***Corresponding author:**

Naoki Fujita

Research and Development Group., Nippon Electric Glass Co., Ltd., 2-7-1 Seiran, Otsu, Shiga 520-8639, Japan

Tel: +81-77-534-1312, Fax: +81-77-534-3572; Email: nfujita@neg.co.jp

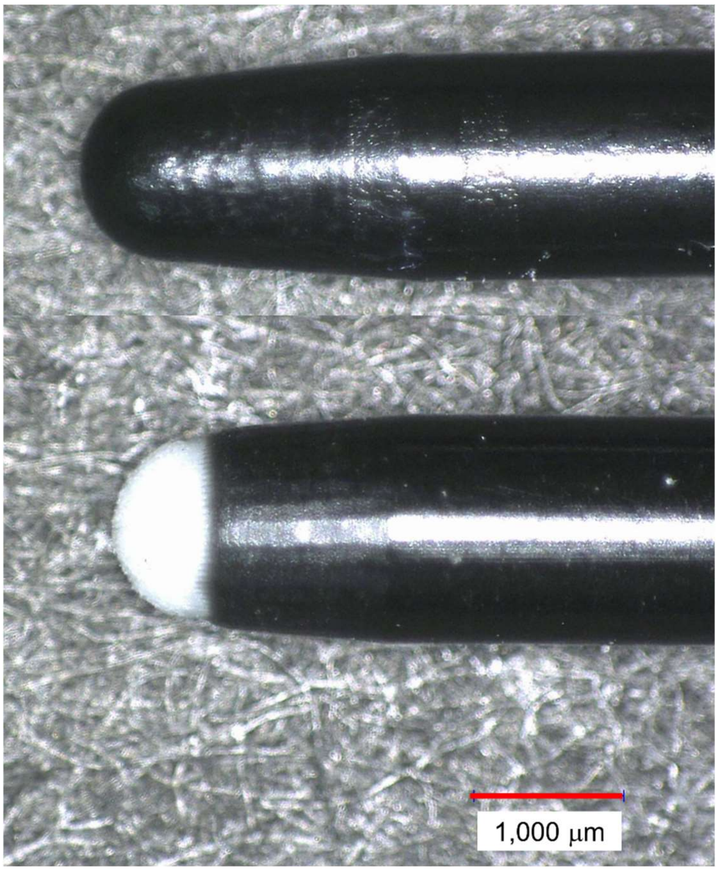

Figure S1. Appearance of the writing-tip specimens (upper: polyacetal, bottom: elastomer).

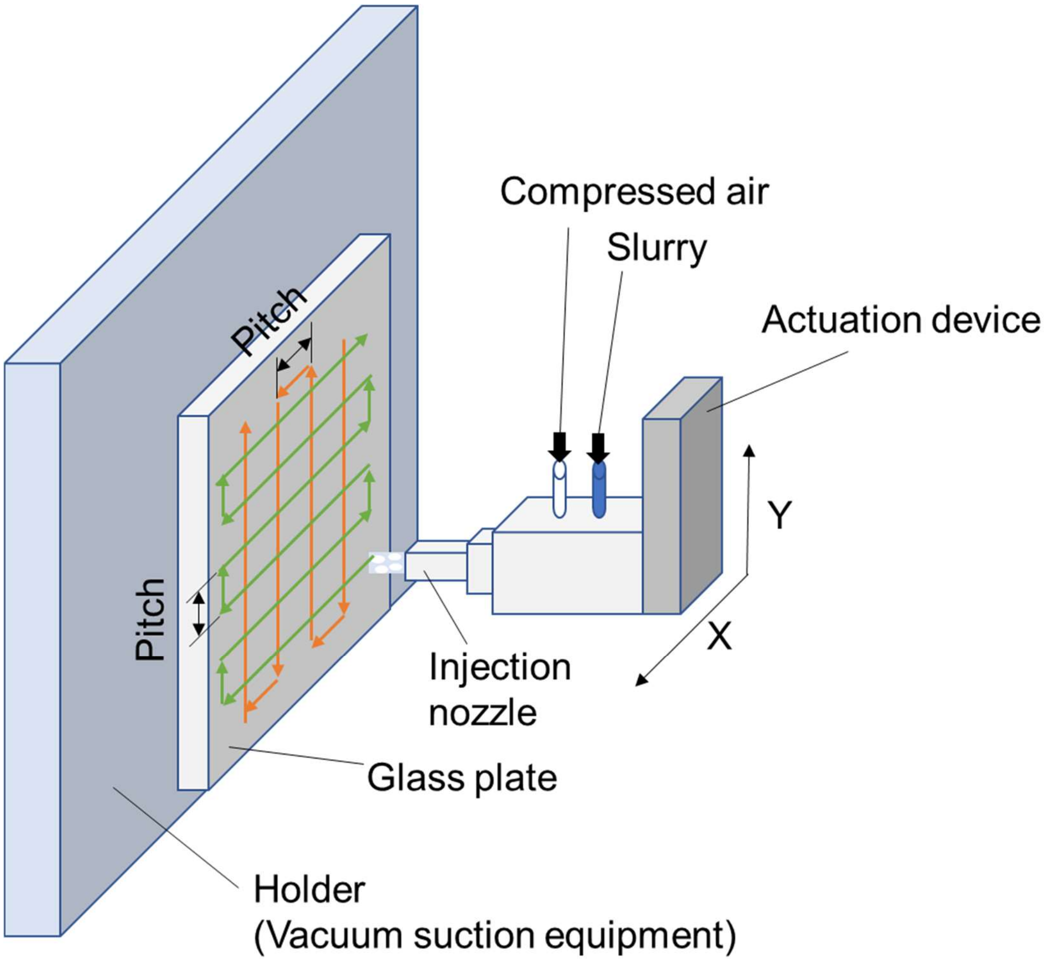

Figure S2. Schematic of the processing of textured glass writing surfaces.

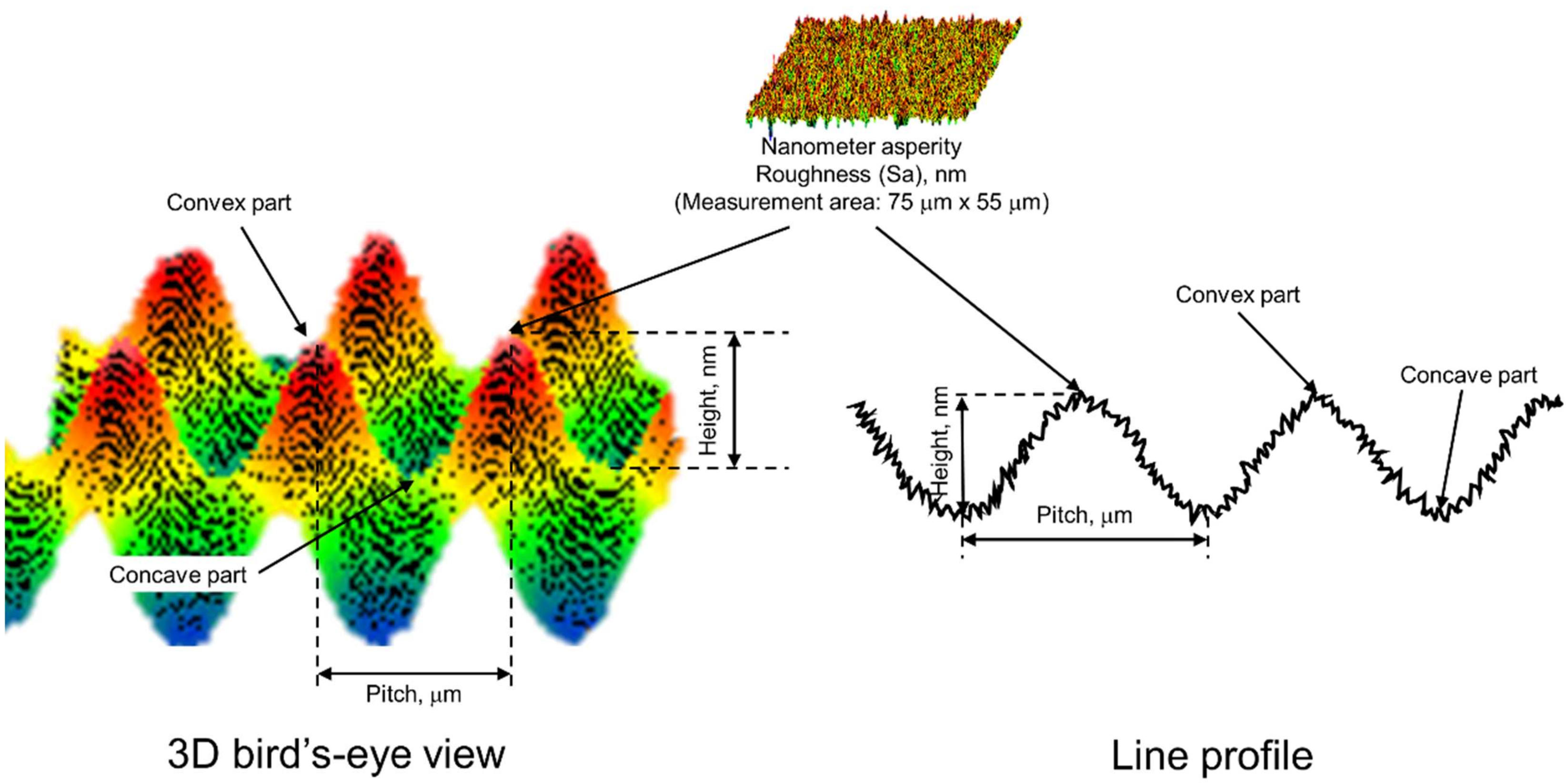

Figure S3. Geometrical parameters of the textured glass surface in 3D bird's-eye view and line profile and image of nanometer sized asperity.

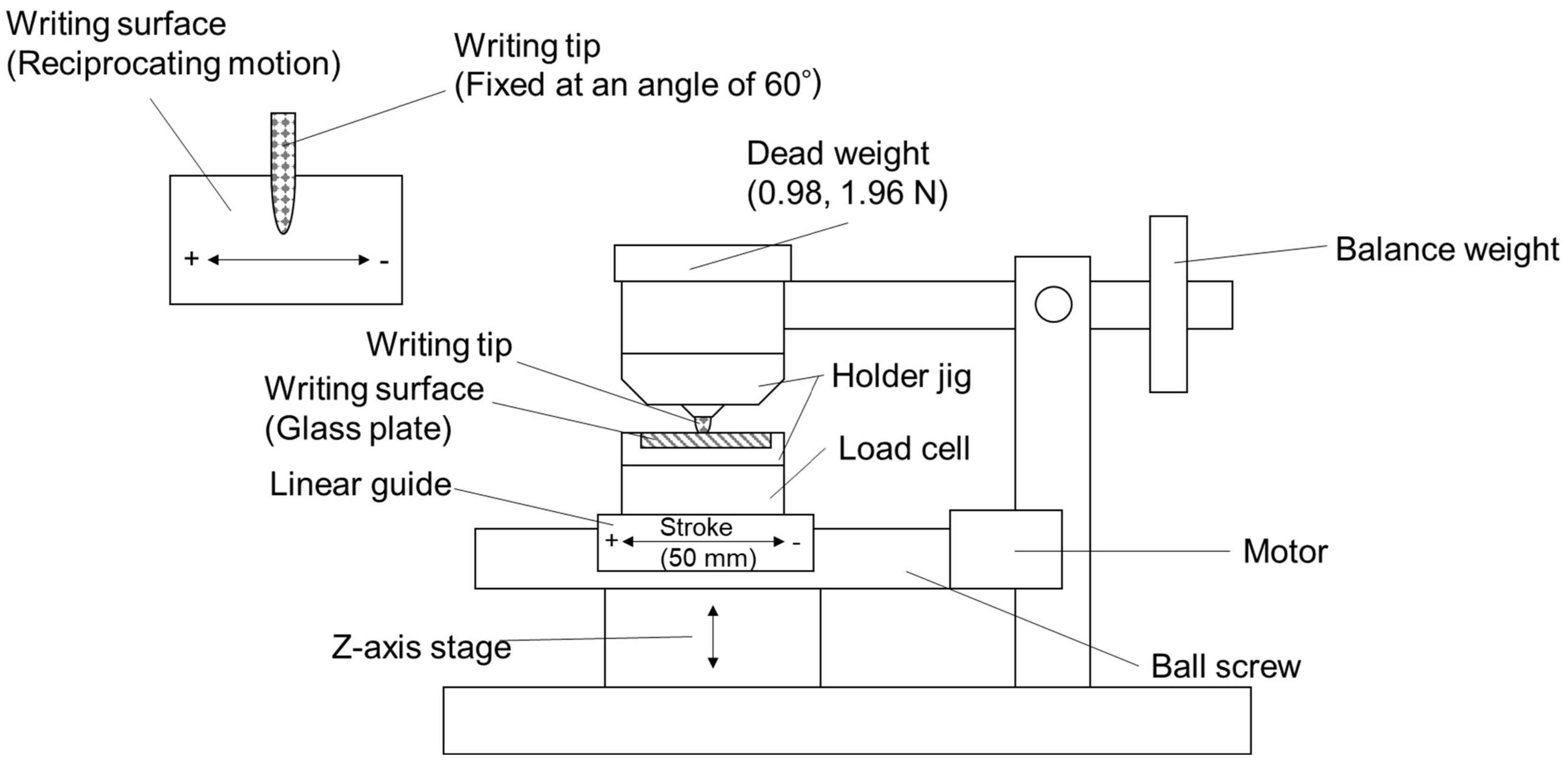

Figure S4. Schematic of the reciprocating motion tests for friction measurement.

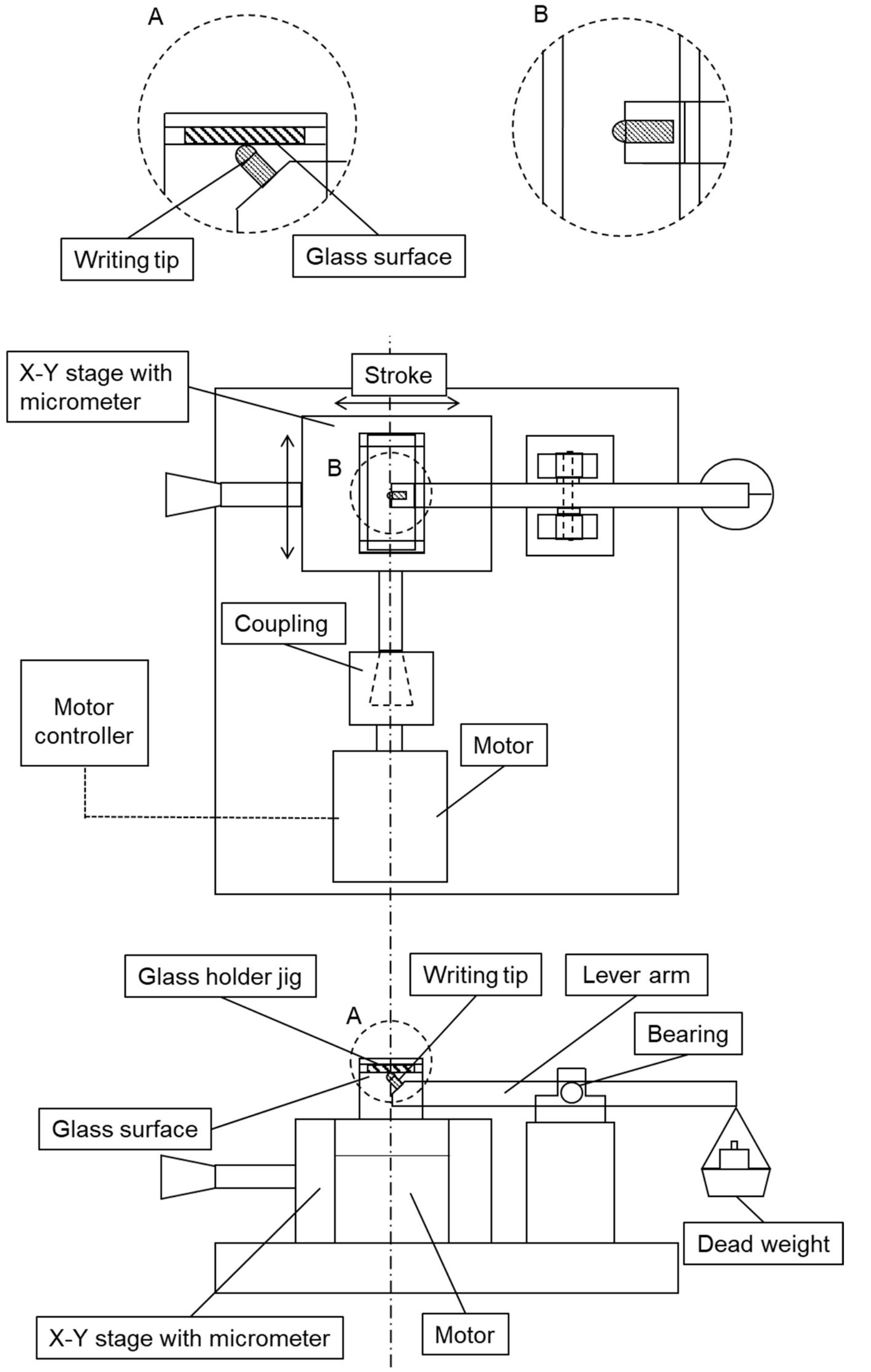

Figure S5. Schematic of contact area observation between the writing tips and the glass surfaces during the sliding motions.
