# Supplementary material for: Friction control of elastic materials on glass by means of textured surfaces
Source: Sci Rep. 2022 Sep 14;12:15423. doi: 10.1038/s41598-022-19338-7 (PMC9474819; doi:10.1038/s41598-022-19338-7)
Supplement: Supplementary file 2 — Supplementary Information 2. [file 41598_2022_19338_MOESM2_ESM.pdf]

Supplementary Videos for

Friction control of elastic materials on glass by means of textured surfaces

Naoki Fujita[1,2,*], Takumi Kinoshita[1], Masaru Iwao[1], Noriaki Masuda[1], and Yoshitaka Nakanishi[3]

[1] Research and Development Group., Nippon Electric Glass Co., Ltd., 2-7-1 Seiran, Otsu, Shiga 520-8639, Japan

[2] Graduate School of Science and Technology, Kumamoto University, 2-39-1 Kurokami, Chuo-ku, Kumamoto 860-8555, Japan

[3] Faculty of Advanced Science and Technology, Kumamoto University, 2-39-1 Kurokami, Chuo-ku, Kumamoto 860-8555, Japan

***Corresponding author:**

Naoki Fujita

Research and Development Group., Nippon Electric Glass Co., Ltd., 2-7-1 Seiran, Otsu, Shiga 520-8639, Japan

Tel: +81-77-534-1312, Fax: +81-77-534-3572; Email: nfujita@neg.co.jp

Supplementary Video 1.

Video corresponding to Fig. 4 (b)-(c). Changes in the contact area between the elastomer and glass surfaces during sliding motion ((a) Stripe pattern with 500 μm spacing and 1,463 nm, (b)flat glass).

Supplementary Video 2.

Video corresponding to Fig. 5 (a)-(b). Changes in the contact area between the polyacetal and glass surfaces during sliding motion ((a) Stripe pattern with 500 μm spacing and 1,463 nm, (b)flat glass).

Supplementary Video 3.

Video corresponding to Fig. 7 (b). Changes in the contact area between the elastomer and lattice-shaped patterned glass surface during sliding motion (pitch: 500 μm, height: 560 nm).
